# The Clinical Value of High-Intensity Signals on the Coronary Atherosclerotic Plaques: Noncontrast T1-Weighted Magnetic Resonance Imaging

**DOI:** 10.3390/ijms17071187

**Published:** 2016-07-21

**Authors:** Shoichi Ehara, Kenji Matsumoto, Kenei Shimada

**Affiliations:** Department of Cardiovascular Medicine, Osaka City University Graduate School of Medicine, Osaka 545-8585, Japan; matsumoto1110@hotmail.co.jp (K.M.); shimadak@med.osaka-cu.ac.jp (K.S.)

**Keywords:** acute coronary syndrome, atherosclerosis, magnetic resonance imaging, plaque, thrombosis, intraplaque hemorrhage

## Abstract

Over the past several decades, significant progress has been made in the pathohistological assessment of vulnerable plaques and in invasive intravascular imaging techniques. However, the assessment of plaque morphology by invasive modalities is of limited value for the detection of subclinical coronary atherosclerosis and the subsequent prediction or prevention of acute cardiovascular events. Recently, magnetic resonance (MR) imaging technology has reached a sufficient level of spatial resolution, which allowed the plaque visualization of large and static arteries such as the carotids and aorta. However, coronary wall imaging by MR is still challenging due to the small size of coronary arteries, cardiac and respiratory motion, and the low contrast-to-noise ratio between the coronary artery wall and the surrounding structures. Following the introduction of carotid plaque imaging with noncontrast T1-weighted imaging (T1WI), some investigators have reported that coronary artery high-intensity signals on T1WI are associated with vulnerable plaque morphology and an increased risk of future cardiac events. Although there are several limitations and issues that need to be resolved, this novel MR technique for coronary plaque imaging could influence treatment strategies for atherothrombotic disease and may be useful for understanding the pathophysiological mechanisms of atherothrombotic plaque formation.

## 1. Introduction

Acute myocardial infarction or sudden cardiac death frequently occurs as the first symptom of coronary diseases, without prodromal angina [1]. Therefore, the prediction or prevention of acute cardiovascular events has become a crucial clinical issue.

The degree of luminal narrowing is used as a marker for high risk plaques [2], but it is widely recognized that plaque composition is likely to be much more clinically significant than luminal narrowing because the arterial lumen is often preserved by positive arterial remodeling [3]. Therefore, the direct evaluation of the arterial wall is an important goal in cardiovascular imaging. From the 1990s onward, pathohistological studies have demonstrated that plaque rupture or erosion of the endothelial surface with subsequent thrombus formation is the most important mechanisms in acute coronary syndromes (ACSs) [4,5,6]. A large lipid-pool, thin-cap fibroatheroma (TCFA), macrophage accumulation, and intraplaque hemorrhage have been identified as the key features of rupture-prone plaques [7]. Over the past several decades, significant progress has been made in the assessment of vulnerable plaques using invasive intravascular imaging, such as intravascular ultrasound (IVUS) [8,9,10], coronary angioscopy [11,12], or optimal coherence tomography (OCT) [13,14,15]. Those vulnerable features in the coronary artery have been confirmed not only in patients who died from the disease but also in patients who survived an ACS. However, the assessment of plaque morphology by invasive modalities is of limited value for the detection of subclinical coronary atherosclerosis and the subsequent prediction or prevention of acute cardiovascular events.

Hence, there is widespread interest in alternative non-invasive modalities, such as magnetic resonance (MR) imaging or computed tomography (CT), to directly visualize the arterial wall and characterize plaque composition. MR imaging is an attractive option because it is performed with magnetic fields and it is a safe and completely non-invasive technique with excellent soft tissue contrast capable of differentiating the plaque components on the basis of their biophysical and biochemical parameters.

In this review, we focus on the accumulated data on vulnerable plaque imaging using MR techniques, and we also introduce the results from our recent studies.

## 2. The Beginning of Coronary Artery Plaque Imaging Based on High-Intensity Signal on Noncontrast T1-Weighted Imaging

With any imaging technique, its most important qualities are the spatial resolution required to visualize the lesion components and good contrast between the various components of the lesions. Recently, MR imaging technology has reached a sufficient level of spatial resolution, which allowed the plaque visualization of large and static arteries such as the carotids and aorta [16,17,18,19,20]. The advent of carotid plaque characterization with noncontrast T1-weighted imaging (T1WI) in MR has facilitated plaque imaging based on the presence of a high-intensity signal (HIS) within the thrombus or intraplaque hemorrhage caused by methemoglobin T1 shortening [6,7,8,9,10,11,12,13,14,15,16,17,18,19,20,21].

However, coronary wall imaging by MR is still challenging due to the small size of coronary arteries, cardiac and respiratory motion, and the low contrast-to-noise ratio between the coronary artery wall and the surrounding structures. Despite these challenges, coronary wall imaging by MR has been successfully applied in patients using breathhold [22,23] or respiratory gating (i.e., free-breathing) techniques [24,25,26,27,28]. It has been demonstrated that MR can measure coronary vessel area, wall thickness, plaque burden, or arterial remodeling [23]. However, the use of MR to identify plaque components in coronary arteries has been limited.

**Figure 1:**
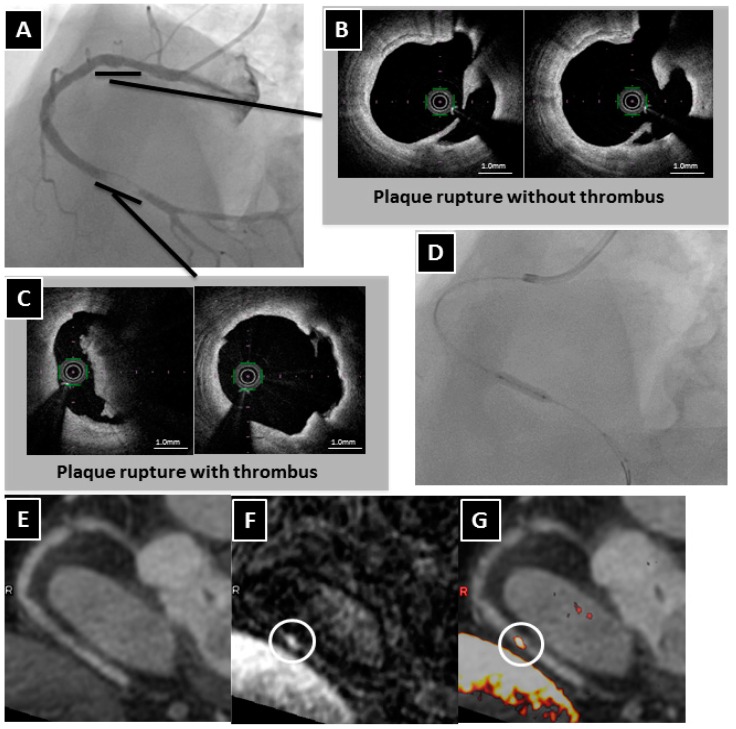
A representative case of a HIS lesion on T1WI associated with an intraluminal thrombus. (**A**) Coronary angiography revealing an intracoronary thrombus identified by the presence of intraluminal filling defects surrounded by contrast agents in the distal right coronary artery (RCA) and an ulceration in the proximal RCA; (**B**) The OCT examination showing a plaque rupture without a thrombus in the proximal RCA; (**C**) In contrast, the culprit lesion in the distal RCA with a plaque rupture with a large intracoronary thrombus; (**D**) Thrombus aspiration and plain old balloon angioplasty (POBA) performed on the culprit lesion; (**E**) Two days later after POBA, whole-heart coronary MR angiography revealing no significant stenosis in the distal RCA; (**F**) Coronary T1WI demonstrating intraluminal HIS on the culprit lesion (circle). However, there is no HIS at the ulceration of the proximal RCA; (**G**) Fused image showing intraluminal HIS in the area corresponding to the culprit lesion (circle). R in panels **E**–**G** indicates right side.

Some investigators have reported that coronary artery HISs on T1WI are associated with a vulnerable plaque morphology [24,25,26,28] and an increased risk of future cardiac events [27]. These coronary plaque images have been obtained while the patients were breathing freely, by using a three-dimensional T1WI, inversion-recovery, gradient-echo technique with fat-suppression. Kawasaki et al. proposed the calculation “the ratio between the signal intensities of coronary plaque and cardiac muscle (PMR)”, which was defined as the highest signal intensity of the coronary plaque divided by the signal intensity of the left ventricular muscle near the coronary plaque [24]. Areas with a PMR >1.0 were defined as HIS in this report. They reported that the typical coronary HIS on T1WI was associated with a high frequency of IVUS-derived low attenuation and positive remodeling, remarkably low CT density, and transient slow-flow phenomena during percutaneous coronary intervention (PCI) [24]. These features seemed to represent vulnerable plaques. Jansen et al. reported that the HIS on T1WI correctly corresponded to the intracoronary thrombus detected by invasive coronary angiography in patients with acute myocardial infarction within 72 h after the initial onset of symptoms [25]. In our study involving a small number of patients, we demonstrated a direct association between coronary HISs on T1WI and the presence of intracoronary thrombus as detected through OCT (Figure 1) [26].

## 3. What Appears as High-intensity Signal on T1-Weighted Imaging in the Coronary Artery? What Is the Best PMR Cutoff Value?

**Figure 2:**
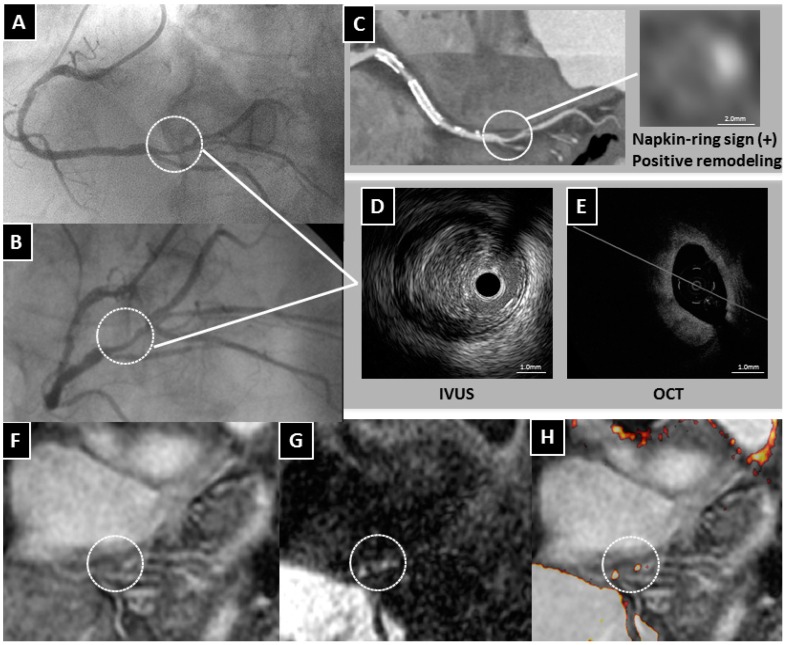
A representative case of an intrawall HIS lesion on T1WI compared with plaque morphology on CT, IVUS and OCT. (**A**,**B**) Coronary angiography revealing severe coronary stenosis (circle) in the distal right coronary artery (RCA); (**C**) Coronary CT angiography showing the napkin-ring sign and positive arterial remodeling; (**D**) The IVUS image showing a low attenuation plaque; (**E**) The OCT examination showing a signal-poor region with irregular high- or low-backscattering borders without thrombus; (**F**) Whole-heart coronary MR angiography showing significant stenosis in the distal RCA (circle); (**G**) Coronary T1WI demonstrating intrawall HIS (circle); (**H**) Fused image showing intrawall HIS (circle) in the area corresponding to the severe stenosis.

**Figure 3:**
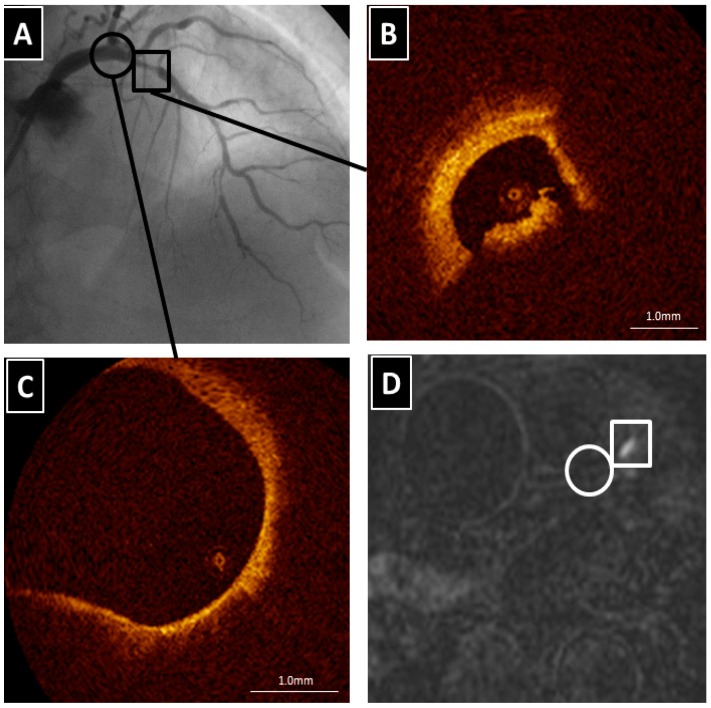
The culprit lesion in the proximal left anterior descending coronary artery (LAD). (**A**) Coronary angiography revealing severe coronary stenosis in the proximal LAD (square) and no significant stenosis in the left main coronary artery (LMCA) (circle); (**B**) The OCT examination showing intracoronary thrombus in the proximal LAD; (**C**) There is a lipid-rich plaque in the LMCA (circle); (**D**) Coronary T1WI demonstrating HIS in the area corresponding to the culprit lesion of the proximal LAD (square). However, no HIS in the LMCA with the lipid-rich plaque is found (circle).

There are two highly controversial topics related to HIS in the coronary artery. First, what appears as a HIS on T1WI in the coronary artery? At this stage, the precise characterization of HISs is not known, because no comparisons with histopathological data have been performed in the coronary artery studies; comparative studies have been done on the carotid artery. Therefore, indirect comparisons have been performed by using a surrogate marker mostly derived from other imaging modalities, such as IVUS or OCT. Recently, Teruo Noguchi et al. reported that coronary artery HISs on T1WI were associated with future cardiac events in patients with mildly atherosclerotic lesions that had not yet caused an acute coronary event or induced cardiac ischemia [27]. It seems unlikely that HISs localized within the vessel wall are associated with thrombus in the subclinical population. We investigated the relationship between localization of HISs on T1WI and plaque morphology detected on OCT in patients with either stable or unstable angina [28]. Areas with a PMR ≤ 1.0 were classified as non-HISs. HISs with a PMR > 1.0 were then classified into two types, according to the localization of HIS, using cross-sectional coronary T1WI. Areas that were localized within the coronary wall when the lumen was identified were defined as intrawall HISs, whereas areas that occupied the lumen when the lumen was not identified were defined as intraluminal HISs. The multivariate analysis revealed that intraluminal HISs were associated with thrombus and intimal vasculature assessed by OCT. In contrast, macrophage accumulation and the absence of calcification were independent factors associated with intrawall HISs (Figure 2). The plaque morphology of the culprit lesions in ACS patients varies from thrombosis with or without plaque rupture to sudden luminal narrowing caused by intraplaque hemorrhage. When previous data are taken together with our findings, one can speculate that coronary intrawall HISs on T1WI may indicate intraplaque hemorrhage associated with inflammation. Some observations have indicated that hemorrhage components appear as signal-poor OCT regions that must be distinguished from lipid necrotic pools. Regrettably, the current imaging techniques, including OCT, do not allow a definitive discrimination between hemorrhage and lipid components, and that is a major issue that will require additional validation studies using histopathological materials from coronary, rather than carotid arteries. In addition, we found that in intrawall HIS lesions the presence of lipid-rich plaques was more frequent than in non-HIS lesions, although these differences were not statistically significant when analyzed by multivariate analysis [28]. Do lipid-rich plaques generate HIS? Although this MR technique uses a fat-suppressed sequence, perivascular fat, which is mainly composed of triglycerides, has a different appearance on MR than the lipids in atherosclerotic plaques. The plaque lipids consist primarily of unesterified cholesterol and cholesterol esters [29]. Therefore, it is not known whether the lipids within the atherosclerotic plaques were successfully suppressed. There is increasing evidence that multiple vulnerable plaques with lipid are present within the whole coronary tree in patients who experience an ACS, even though it may be a single localized culprit lesion that caused the acute cardiovascular event [30]. However, most lipid pools do not generate HISs except at the culprit lesion (Figure 3). The proportion, age, and volume of methemoglobin based on the presence of vulnerable complex plaques may determine the PMR values.

The second controversial topic is the fact that several MR studies used different PMR cutoff values to detect HIS, so there is no consensus on which cutoff is best for risk stratification. Recently, two studies by Noguchi et al. demonstrated that the optimal PMR cutoff values for predicting future cardiac events and myocardial injury during elective PCI, defined as an increase in serum troponin T levels, were of 1.4 and 1.3, respectively [27,31]. At this stage, it is not known which PMR cutoff value is best or whether there is a need to determine the cutoff values. In our unpublished data, the PMR increased in proportion to the accumulation of the number vulnerable plaque features, such as intraluminal thrombus, lipid-rich plaque, plaque rupture, macrophage accumulation, and intimal vasculature. HISs with a higher PMR are likely to represent more vulnerable plaques. Future studies are needed to clarify the significance of PMR values on T1WI.

## 4. The Clinical Implication of High-Intensity Signal in Coronary Atherosclerotic Plaques

What is the clinical implication of detecting HIS on noncontrast T1WI in MR in coronary atherosclerotic plaques? One of the goals of researchers is to investigate whether the presence of HIS on T1WI in subclinical coronary atherosclerosis is associated with the subsequent development of acute cardiovascular events. Noguchi et al. demonstrated that a PMR cutoff value of 1.4 was best for identifying vulnerable coronary plaques associated with future cardiac events, including nonfatal ST-segment elevation myocardial infarction, high-sensitivity cardiac troponin T-positive unstable angina pectoris or non-ST-segment elevation myocardial infarction, and ischemia-driven PCI due to progressive angina pectoris [27]. Moreover, their stratified analysis using PMR values of 1.0 and 1.4 revealed that the incidence of cardiac events was well differentiated: 25.8% for PMR ≥ 1.4, 8.4% for PMR 1.0–1.4, and 1.1% for PMR < 1.0. Interestingly, of the segments with plaques with PMRs ≥ 1.4, 17% were associated with coronary events, which developed in 51% of the segments in the first 12 months.

Moreover, some studies have demonstrated that the presence of HIS on noncontrast T1WI has the potential to predict a PCI-related myocardial injury, which is associated with worse short-term and long-term clinical outcomes [31,32,33]. Although the etiology of PCI-related myocardial injury is a multifactorial phenomenon, the predominant mechanism involves the distal embolization of atheromatous or thrombotic materials, and results from the mechanical fragmentation of the culprit plaque during PCI [34]. Asaumi et al. examined the relationship between HISs on T1WI and PCI-related myocardial injury, which is manifested by the elevation of cardiac troponin, in patients undergoing elective PCI [31]. They reported that the optimal PMR cutoff value for predicting PCI-related myocardial injury was 1.3, and the sensitivity and specificity were 67% and 86%, respectively. Hoshi et al. also reported that in patients with stable angina pectoris undergoing elective PCI, the PMR cutoff value of 1.44 predicted PCI-related myocardial injury, and the sensitivity and specificity were 78% and 82%, respectively [32]. In our study, which employed distal protection devices, we investigated predictors of the filter no-reflow (FNR) phenomenon during PCI by using multimodality, such as HIS on T1WI, plaque composition by using OCT, and serum biomarkers, in patients with either stable or unstable angina [33]. Our multivariate analysis revealed that only the presence of HISs with PMR > 1.85 remained an independent predictor of the FNR phenomenon, and the sensitivity and specificity were 65% and 93%, respectively. It is unclear why there are differences in PMR cutoff values for predicting cardiac events. In our study involving patients with unstable angina pectoris, FNR occurred at a high rate (40%) compared with the rate of PCI-related myocardial injury (26%) reported in a previous study [31]. HISs with a higher PMR are likely to represent more vulnerable plaques, and thus the occurrence of FNR might be more closely associated with vulnerable plaque morphology and plaque volume with a higher PMR than that of PCI-related myocardial injury assessed by troponin. The differences in study population or outcomes might be associated to the different PMR cutoff values.

A noninvasive imaging technique capable of identifying not only the presence of a thrombus or hemorrhage but also its stage of development would be invaluable. It has already been established that cerebral hemorrhage staging can be accurately assessed by MR using multicontrast images. Despite the existence of well-defined criteria for staging cerebral hemorrhages, few reports are available regarding the staging of an intraluminal thrombus or intraplaque hemorrhages. Recently, Tan et al. revealed that MR imaging was a precise and reproducible method for distinguishing an acute ipsilateral recurrent deep vein thrombosis from an at least six-month-old chronic residual thrombus in the leg veins, when recurrence was not suspected [35]. Moreover, Chu et al. have shown that multicontrast MR images can detect and classify a carotid intraplaque hemorrhage into three stages: fresh (<1 week), recent (1–6 weeks), and old (>6 weeks) [36]. If HIS on T1WI can be shown to be limited to a fixed term, its presence could be used to accurately identify recent plaque thrombosis or hemorrhage. This information may have several novel clinical implications in the field of PCI, including the prediction or prevention of no-flow phenomena or the aging of a chronic total occlusion. The precise assessment of recent plaque thrombosis or hemorrhage in the coronary occlusion site may influence procedural success rates for chronic total occlusions. Furthermore, although ACS patients with the high-risk should be considered for early invasive intervention, the differential diagnosis and treatment of the remaining patients is challenging in emergency triage. A noninvasive thrombus-detection technique would be useful for further risk stratification and for obtaining prognostic information in patients with coronary artery disease.

Finally, the extent of intraplaque hemorrhage corresponded positively to the size of necrotic core, and the development of hemorrhage resulted in plaque volume expansion and subsequent plaque rupture [37]. If coronary intrawall HISs on T1WI represent intraplaque hemorrhage, early identification of patents with hemorrhage may prove invaluable in optimizing management to minimize future cardiovascular events. Noguchi et al. reported that statin therapy reduced the PMR values, as well as low-density lipoprotein cholesterol and high-sensitivity C-reactive protein levels in patients with coronary artery disease [38]. If statin therapy not only modifies plaque morphology and makes it more stable, but also accelerates the degradation of methemoglobin, that could be invaluable for treatment strategies for atherosclerosis.

## 5. Limitations and Issues to Be Resolved in the Future

Several limitations should be mentioned in this study field. First, the major issue is that there was no evidence based on pathohistological findings. Therefore, the previous results should be interpreted with caution. Second, when an inversion-recovery gradient-echo sequence is used for the T1WI, issues with spatial resolution and partial volume effect could provide artifacts that look like HIS. This MR technique overcomes many of the difficulties associated with conventional techniques that generate a signal based on flowing blood. With T1WI, signal generation does not rely on flowing blood because it uses a non-slice-selective inversion recovery pulse for the black-blood method. Therefore, the image interpretation requires only the detection of a high signal, beyond the high-resolution display of vessel walls [21]. Although theoretically the T1WI technique is unaffected by blood flow, it is not known whether this supposition applies to actual clinical images. There is a possibility that the HIS might result from a gap in a null point. Future studies are needed to verify whether flow disturbance cause artifact like a HIS. Third, contrast agents such as gadopentic acid (Gd-DTPA), attached to specific imaging probes targeted to biochemical and cellular markers of atherosclerotic plaque vulnerability, may be useful for plaque characterization [39]. Finally, previous coronary plaque imaging studies on MR used only a single-contrast sequence as T1WI [24,25,26,27,28,31,32,33,38]. The multi-contrast high-resolution protocol is ideal since they generate a wide range of contrast for the individual plaque characterization. However, the acquisition of multi-contrast images is time-consuming, especially in conjunction with the need for high spatial resolution.

## 6. Conclusions

To determine which plaque features pose a higher risk for future cardiovascular events, we need a noninvasive imaging tool that can identify high-risk plaque features. MR imaging has the potential to identify thrombus and distinguish intraplaque hemorrhage from other plaque components. Because this MR technique has a very short history and no comparisons with histopathological data or multicenter randomized trials have been carried out, many more studies will be needed before this method could be considered to be applied in the clinical arena. Although there are several limitations and issues that need to be resolved, this novel MR technique for coronary plaque imaging could influence treatment strategies for atherothrombotic disease and may be useful for understanding the pathophysiological mechanisms of atherothrombotic plaque formation.

## Abbreviations

The following abbreviations are used in this manuscript:

ACS	acute coronary syndrome
TCFA	thin-cap fibroatheroma
IVUS	intravascular ultrasound
OCT	optical coherence tomography
MR	magnetic resonance
CT	computed tomography
T1WI	T1-weighted imaging
HIS	high-intensity signal
PMR	the ratio between the signal intensities of coronary plaque and cardiac muscle
PCI	percutaneous coronary intervention
FNR	filter no-reflow
